# The Association of Preoperative Trail Making Tests With Postoperative Delirium

**DOI:** 10.7759/cureus.44171

**Published:** 2023-08-26

**Authors:** Mrityunjay Mundu, Ram Chandra Besra, Niranjan Mardi, Saurav K Singh, Puja Pallavi, Ajay K Bakhla

**Affiliations:** 1 Surgery, Rajendra Institute of Medical Sciences, Ranchi, IND; 2 General Surgery, Rajendra Institute of Medical Sciences, Ranchi, IND; 3 Surgery, Sheikh Bhikhari Medical College, Hazaribagh, IND; 4 Psychiatry, Narayan Medical College and Hospital, Rohtas, IND; 5 Psychiatry, Rajendra Institute of Medical Sciences, Ranchi, IND

**Keywords:** preoperative, trail making test, postoperative delirium, cognitive impairment, anxiety

## Abstract

Aims

The aim of the present study was to investigate the preoperative Trail Making Test (TMT) and its association with postoperative delirium.

Materials and methods

This cross-sectional, observational study consisted of 51 patients admitted to the surgical ward for any planned operative procedure. Consenting patients provided their sociodemographic information, and the Hospital Anxiety and Depression Scale (HADS), Montreal Cognitive Assessment (MoCA) test, and Trail Making Test (TMT) were applied.

Results

A total of 51 patients (66.7% male and 33.3% female) were categorized as the “normal” group (n=34), completing TMT in time, and the “slow” group (n=17). The mean age was 45.05 ± 13.69 for the normal group and 44.29 ± 10.95 for the slow group. The HADS score mean was 15.02 ± 9.52 and 11.64 ± 5.73, respectively, for these two groups (t = -1.577; degrees of freedom {df} = 47.11; p = 0.121). However, the “normal” group scored significantly higher MoCA scores in comparison to the slow group (26.35 ± 1.06 and 24.29 ± 1.10, respectively) (t = -6.410; df = 49; p = 0.000).

Conclusions

The study shows that the TMT can indicate effectively the cognitive decline in preoperative patients, which predicts postoperative delirium.

## Introduction

In a systematic review, different rates of incidence for postoperative delirium were found for surgeries of different organs or systems, the lowest incidence was 10% with urologic surgeries, and 26% was the overall incidence rate [1]. The incidence of delirium after noncardiac surgical procedures was found to be 18.6% (95% CI: 16.4-20.8) among the elderly population [2].

Several risk factors have been identified for delirium in post-surgical patients across various studies, including increasing age (over 80 years), four or more comorbid conditions, impaired senses, impaired functional physical status, severe illness, infection, and alcohol use [3-7]. Many screening tools and assessment tools are currently available for delirium in post-surgical patients.

A recent large systematic review of prediction models for delirium reported that impaired preexisting cognitive function is the most significant factor in precipitating delirium [7]. The assessment of cognitive function is usually not considered in surgical setup, but as the presence of cognitive dysfunction may precipitate delirium, assessment and preventive measures may be important to predict delirium. Various neuropsychological tests are currently used to screen for cognitive impairments, such as tests for attention, temporal orientation, and short-term memory, as well as the months of the year backward test, the intersecting pentagons copying test [8], and the digit span backwards test [9]. There is evidence that simple bedside tests of various neuropsychological functions (e.g., attention, concentration, and visuospatial ability) have diagnostic implications for neurocognitive disorders, including delirium [10].

In a systematic review and meta-analysis [11] of postoperative cognitive dysfunction, there was no consensus about the diagnostic utility of neuropsychological tests. Still, the most common tests were the mini-mental state examination, the digit span task, Part A of the Trail Making Test (TMT), and the digit symbol substitution test, which are used up to seven days postoperatively. However, most of these studies focused on general medical conditions among geriatric age range populations. Therefore, the aim of this study was to assess preoperative cognitive slowness as measured by the TMT as an indicator of postoperative delirium.

## Materials and methods

The aim of the present study was to assess preoperative cognitive function and determine whether any subclinical cognitive impairment posed a risk for postoperative delirium. We conducted this study at the various surgical wards of a tertiary care medical college hospital after the institutional review board approved the study protocol. All measures were taken to ensure that no personal identifying information of the patients could be revealed. This was a brief observational study carried out from July 2020 to December 2022.

Subjects

The study subjects were patients who were admitted to surgical wards of our tertiary care center for any elective planned surgery. The inclusion criteria were those of either gender, those who were within the age group of 18-65 years, those who consented to participate in the study, and those who were educated up to class 10 or above. The exclusion criteria were those having emergency surgery, those diagnosed with dementia or any other amnestic disorders, patients with alcohol or other substance dependence, patients with head injury or seizure disorder, and those who scored less than 26 on a preoperative Montreal Cognitive Assessment (MoCA).

Tools

Several tools were used in this study. First, we used a sociodemographic data sheet to record the age and gender of the patients, as well as clinical information such as years of education, marital status, occupation, and habitat. Second, we used the well-validated Hospital Anxiety and Depression Scale (HADS) [12] to assess anxiety and depression among hospitalized patients. The HADS consists of 14 questions: seven scoring anxiety and seven scoring depression. We asked patients to read each question and place a tick against the reply that came closest to how they had been feeling that day. As each answer was scored 0, 1, 2, or 3, the possible range of scores was 0-21, with higher scores indicating greater levels of anxiety. A score of 0-7 is considered normal, a score of 8-10 is borderline abnormal, and a score of 11-21 is an abnormal case. Third, we used the MoCA [13], which is considered a very sensitive test for cognitive impairment. MoCA score ranges between zero and 30, with lower scores indicating worse cognitive functioning. The MoCA items are categorized into six domains: memory, executive functioning, attention, language, visuospatial, and orientation. The administration of the MoCA was done by trained specialized nurses assisted by a professional interpreter.

Finally, we used the TMT [14], which is an executive measure of sequencing and cognitive flexibility. The TMT (Part A) requires the subject to rapidly sequence a straightforward series. The TMT Part B is a more difficult cognitive flexibility task requiring the subject to follow a sequential pattern while shifting cognitive sets, reflecting executive functioning, although other cognitive abilities (e.g., psychomotor speed and visual scanning) are also necessary for the successful completion of the task [15]. The scoring for this test is the time in seconds required to complete it. The test is discontinued at 300 seconds if the subject is not able to complete it in that period of time [16].

Procedure

This was a cross-sectional observational study. Patients who were admitted to our surgical wards for any elective planned surgery were assessed for inclusion and exclusion criteria; on qualification, they were asked to fill out the sociodemographic data sheet, or the questions were asked verbally, and the data sheet was filled out by the investigators. The anxiety subset of the HADS was applied to all subjects and recorded. Similarly, the MoCA and TMT were applied to each patient on the evening before their elective planned surgery. Every day postoperatively for one week, each patient’s condition was assessed clinically and/or via the MoCA for any postoperative delirium.

Statistical analysis

We statistically analyzed the collected data of all patients using the Statistical Package for Social Sciences version 10.0 (SPSS Inc., Chicago, IL). The sample was categorized on the basis of TMT speed of completion as the normal speed group and the slower speed group, as based on age and gender-specified norms. The data analysis included sociodemographic characteristics, for which we calculated means and standard deviations for all continuous variables and percentages for all categorical variables across groups and statistically compared them with the chi-square test or Student’s t-test. Furthermore, we compared the mean scores of total HADS, mean scores of the HADS depression and anxiety subscales, and MoCA scores across groups. We used the parametric Student’s t-test again to determine whether differences existed between the groups. Finally, we found a set of patients who developed postoperative delirium and compared it with the chi-square test with the number of patients who did not develop postoperative delirium. We set the levels of statistical significance and high statistical significance at p ≤ 0.05 and p < 0.001, respectively.

## Results

A total of 51 patients (66.7% male and 33.3% female) were included in the study, with 86.3% married, 76.5% belonging to a rural background, and 64.7% self-employed and of a low socioeconomic class. The mean age of the complete sample was 44.80 years, and the mean duration of education was 11.29 years, as we excluded those with education levels below class 10. We further categorized the total sample on the basis of normative data [17] stratified by age and education. Thirty-four participants with normal TMT speeds comprised the first group, which we named the “normal group,” whereas the remaining 17 participants had slow TMT speeds, which we named the “slow group.” Table 1 summarizes the sample characteristics across these two categorizations. The mean age of the complete sample was 44.93 years (45.05 ± 13.69 years for the normal group and 44.29 ± 10.95 years for the slow group). The ages of the two groups were statistically similar in the context of mean age. Similarly, education and the duration of illness were comparable between these two groups (Table 1).

**Table 1:** Sociodemographic profile of the sample grouped as normal or slow on Trail Making Tests df: degrees of freedom

Variable		Normal (n = 34)	Slow (n = 17)	t/chi-square	df	p
Age (years)		45.05 ± 13.69	44.29 ± 10.95	200	49	0.842
Education		11.26 ± 1.65	11.35 ± 1.96	168	49	0.867
Gender	Male	25	9	2.162	1	0.141
	Female	9	8			
Marital status	Married	27	17	4.057	1	0.044
	Unmarried	7	0			
Habitat	Rural	23	16	4.413	1	0.036
	Urban	11	1			
Occupation	Employed	12	6	0.000	1	1.000
	Self-employed	22	11			

The mean anxiety score of the total sample was found to be 6.54, which suggested a normal degree of anxiety for the whole sample. There was no significant difference between patients of the normal and slow groups, with mean anxiety scores of 7.23 ± 5.39 and 5.17 ± 4.75, respectively (t = -1.334; degrees of freedom {df} = 49; p = 0.188) (Table 2).

**Table 2:** Comparison of mean anxiety and depression scores among the normal and slow groups MoCA, Montreal Cognitive Assessment; HADS, Hospital Anxiety and Depression Scale; df, degrees of freedom

	Normal (n = 34)	Slow (n = 17)	t	df	Significance (two-tailed)
Mean anxiety score	7.23 ± 5.39	5.17 ± 4.75	-1.334	49	0.188
Mean depression score	7.79 ± 4.57	6.47 ± 2.62	-1.311	47.90	0.196
HADS total	15.02 ± 9.52	11.64 ± 5.73	-1.577	47.11	0.121
MoCA score	26.35 ± 2.06	24.29 ± 2.10	-6.410	49	0.000

The mean depression scores for the normal and slow groups were 7.79 ± 4.57 and 6.47 ± 2.62, respectively (t = -1.311; df = 47.90; p = 0.196). The mean total HADS scores for the normal and slow groups were 15.02 ± 9.52 and 11.64 ± 5.73, respectively (t = -1.577; df = 47.11; p = 0.121). Interestingly, a statistically significant difference was found in mean MoCA score across the two groups, where the normal group scored significantly higher (26.35 ± 1.06) than the slow group (24.29 ± 1.10) (t = -6.410; df = 49; p = 0.000) (Table 2). A total of seven patients, one from the normal group (3%) and six from the slow group (54.54%), developed postoperative delirium, as assessed between postoperative days 1 and 5 (chi-square = 10.018; df = 1; p = 0.002) (Table 3). 

**Table 3:** Cross table of Trail Making Test speed and the development of postoperative delirium df: degrees of freedom

	Intact (n = 44)	Delirium (n = 7)	Chi-square	df	Significance (two-tailed)
Normal	33	1	10.018	1	0.002
Slow	11	6			

## Discussion

In this study, we attempted to assess preoperative anxiety, depression, and cognitive status, as well as their impact on the development of postoperative delirium. We used the HADS because it is a simple, short, and quick test that is easy to explain to the patient and reliable for measuring anxiety and depression [18]. We used the MoCA to measure preoperative cognitive status and postoperative delirium. We also used a single measure of the TMT as a time-based task completion test.

A large review reported postoperative delirium rates of 9%-87% depending on the age of the patient and the type of surgery [19]. Our study found an incidence of seven cases of delirium among a sample size of 51 (13.7%) during the postoperative period. There is much scientific literature about preoperative anxiety and depression that are described in relation to various operative procedures and illnesses. These psychological phenomena are considered complex subjective reactive responses influenced by many factors, including the patient’s temperament and their understanding (or lack thereof) of their illness and the proposed surgery. In this context, anxiety is considered the emotion of fear mixed with feelings of worry and apprehension [20]. A systematic review found that poor performance in all cognitive domains was significantly associated with incident delirium, with the exception of perception. Most of the existing screening tools had varying sensitivity [21]. No rapid screening tools had been validated in the surgical populations; however, the TMT may be used as a simple screening tool to detect at-risk persons, for whom a detailed assessment may be planned.

There was borderline anxiety and depression among preoperative patients; the mean anxiety and depression scores were 6.54 and 7.35, respectively, for a complete sample of 51. Impaired performance on cognitive tests was a risk factor for delirium in our patients, which agrees with data from other studies; however, these studies used different tools to identify cognitive functioning, such as interviews, Activities of Daily Living (ADL) Scale, Blessed Dementia Rating Scale, delirium symptom interview, mini-mental state examination, Confusion Assessment Method (CAM), and Abbreviated Mental Test (AMT); all these measures are used to detect cognitive deficits [4,6,22,23]. The MoCA is a reliable and valid instrument to assess cognitive function. We found mean MoCA scores of 24.29 ± 1.10 and 26.35 ± 1.06 for the slow and normal groups, respectively. Therefore, our results showed that the underlying cognitive dysfunction predisposes surgical patients to postoperative delirium. However, this cognitive dysfunction may be a symptom of depression or anxiety, mild dementia, or mild cognitive impairment, or it may be overlapping comorbid conditions. Although the MoCA is easy to administer in very cooperative patients, the TMT is quick and simple and convenient in preoperative assessment, especially in less cooperative patients. Our study indicates that the TMT can effectively assess cognitive decline in preoperative patients, which is predictive of postoperative delirium. Until now, there is a lack of validated rapid screening for the risk of delirium among the surgical population; however, the TMT can at least be used as a simple screening tool to detect at-risk persons. A detailed assessment may be adequately planned for persons showing deficits in TMT performance. Many preventive measures should be planned to avoid the occurrence of delirium, and prompt management may be done.

In this study, we attempted to identify patients at risk of developing postoperative delirium with a simple measure (i.e., TMT). However, there were limitations to this study, including small sample sizes and unspecified patient diagnoses and surgical procedures. This study also lacks parallel validated cognitive assessment tool to validate the findings by the TMT. Further studies should be planned with a larger sample size, including a comparative study of the TMT with various other neuropsychological tests, with patients of specific diagnoses and surgical procedures.

## Conclusions

In this study, we aimed to determine whether cognitive slowness, as assessed by the TMT, was useful in predicting postoperative delirium. We found that by using the TMT, 33% of preoperative patients were classified as cognitively slow, among which 54.5% of patients developed postoperative delirium in comparison to 3% among cognitively normal persons. Thus, the TMT can effectively indicate cognitive decline or slowness in preoperative patients and predict postoperative delirium.

## References

[1] Igwe EO, Nealon J, O'Shaughnessy P (2023). Incidence of postoperative delirium in older adults undergoing surgical procedures: a systematic literature review and meta-analysis. Worldviews Evid Based Nurs.

[2] Gong XY, Hou DJ, Yang J (2023). Incidence of delirium after non-cardiac surgery in the Chinese elderly population: a systematic review and meta-analysis. Front Aging Neurosci.

[3] Gustafson Y, Berggren D, Brännström B, Bucht G, Norberg A, Hansson LI, Winblad B (1988). Acute confusional states in elderly patients treated for femoral neck fracture. J Am Geriatr Soc.

[4] Marcantonio ER, Flacker JM, Michaels M, Resnick NM (2000). Delirium is independently associated with poor functional recovery after hip fracture. J Am Geriatr Soc.

[5] Baker BR, Duckworth T, Wilkes E (1978). Mental state and other prognostic factors in femoral fractures of the elderly. J R Coll Gen Pract.

[6] Duppils GS, Wikblad K (2000). Acute confusional states in patients undergoing hip surgery. a prospective observation study. Gerontology.

[7] Lindroth H, Bratzke L, Purvis S (2018). Systematic review of prediction models for delirium in the older adult inpatient. BMJ Open.

[8] O'Regan NA, Maughan K, Liddy N (2017). Five short screening tests in the detection of prevalent delirium: diagnostic accuracy and performance in different neurocognitive subgroups. Int J Geriatr Psychiatry.

[9] Leung JL, Lee GT, Lam YH, Chan RC, Wu JY (2011). The use of the digit span test in screening for cognitive impairment in acute medical inpatients. Int Psychogeriatr.

[10] Leonard M, O'Connell H, Williams O (2016). Attention, vigilance and visuospatial function in hospitalized elderly medical patients: relationship to neurocognitive diagnosis. J Psychosom Res.

[11] van Sinderen K, Schwarte LA, Schober P (2020). Diagnostic criteria of postoperative cognitive dysfunction: a focused systematic review. Anesthesiol Res Pract.

[12] Zigmond AS, Snaith RP (1983). The Hospital Anxiety and Depression Scale. Acta Psychiatr Scand.

[13] Nasreddine ZS, Phillips NA, Bédirian V (2005). The Montreal Cognitive Assessment, MoCA: a brief screening tool for mild cognitive impairment. J Am Geriatr Soc.

[14] Reitan RM (1955). The relation of the trail making test to organic brain damage. J Consult Psychol.

[15] Ashendorf L, Jefferson AL, O'Connor MK, Chaisson C, Green RC, Stern RA (2008). Trail Making Test errors in normal aging, mild cognitive impairment, and dementia. Arch Clin Neuropsychol.

[16] Spreen O, Strauss E (1998). A compendium of neuropsychological tests.

[17] Tombaugh TN (2004). Trail Making Test A and B: normative data stratified by age and education. Arch Clin Neuropsychol.

[18] Kolawole IK, Bolaji BO (2002). Reasons for cancellation of elective surgery in Ilorin. Niger J Surg Res.

[19] Whitlock EL, Vannucci A, Avidan MS (2011). Postoperative delirium. Minerva Anestesiol.

[20] Bjelland I, Dahl AA, Haug TT, Neckelmann D (2002). The validity of the Hospital Anxiety and Depression Scale. An updated literature review. J Psychosom Res.

[21] Ghezzi ES, Ross TJ, Sharman R (2022). The neuropsychological profile of delirium vulnerability: a systematic review and meta-analysis. Neurosci Biobehav Rev.

[22] Galanakis P, Bickel H, Gradinger R, Von Gumppenberg S, Förstl H (2001). Acute confusional state in the elderly following hip surgery: incidence, risk factors and complications. Int J Geriatr Psychiatry.

[23] Sato N, Sanuki M, Matsumoto C, Inoue K, Yuge O (2000). Perioperative temporal profile of cognitive function in elderly patients undergoing hip surgery. J Geriatr Psychiatry Neurol.

